# Supplementary material for: Concomitant Activation of OSM and LIF Receptor by a Dual-Specific hlOSM Variant Confers Cardioprotection after Myocardial Infarction in Mice
Source: Int J Mol Sci. 2021 Dec 29;23(1):353. doi: 10.3390/ijms23010353 (PMC8745562; doi:10.3390/ijms23010353)
Supplement: Supplementary file 1 [file ijms-23-00353-s001.zip › ijms-1515190-supplementary.pdf]

Supplementary Materials

Concomitant activation of OSM and LIF receptor by a dual-specific hlOSM variant confers cardioprotection after myocardial infarction in mice

Holger Lörchner[1,5,†], Juan M. Adrian-Segarra[1,†], Christian Waechter[1,3], Roxanne Wagner[1], Maria Elisa Góes[1], Nathalie Brachmann[1], Krishnamoorthy Sreenivasan[1], Astrid Wietelmann[4], Stefan Günther[1], Nicolas Doll[2], Thomas Braun[1,5,*] and Jochen Pöling[1,2,5,*]

[1] Department of Cardiac Development and Remodeling, Max Planck Institute for Heart and Lung Research, 61231 Bad Nauheim, Germany
[2] Department of Cardiac Surgery, Schüchtermann-Clinic, Ulmenallee 5 -11, 49214 Bad Rothenfelde, Germany
[3] Department of Cardiology, University Hospital Marburg, Baldingerstraße, 35043 Marburg, Germany
[4] MRI and µ-CT Service Group, Max Planck Institute for Heart and Lung Research, 61231 Bad Nauheim, Germany
[5] German Centre for Cardiovascular Research (DZHK), Partner site Rhein-Main, Frankfurt am Main, Germany
[†] both authors contributed equally
[*] corresponding authors

e-mail: Thomas.Braun@mpi-bn.mpg.de

e-mail: Jochen.Poeling@mpi-bn.mpg.de

Tel. +49(0)6032-705 1101, Fax +49(0)6032-705 1104

Short Title: Cardioprotection via concomitant activation of OSMR and LIFR

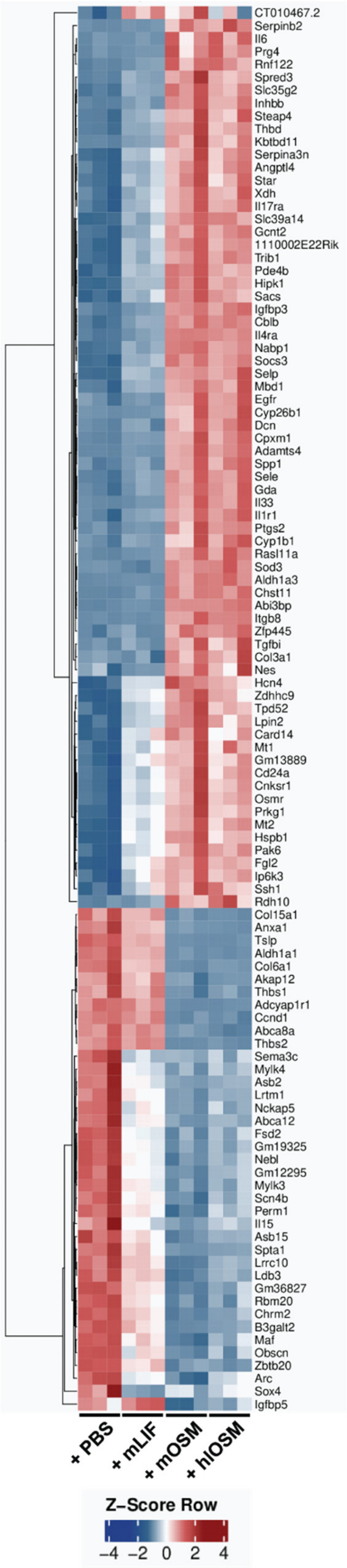

Supplementary Figure S1: Heatmap of differentially expressed genes in PBS– versus cytokine–treated murine cardiomyocytes. Enriched (red) and decreased (blue) genes in mLIF–, mOSM– and hIOSM–treated versus PBS–-treated cardiomyocytes are shown (n=3). The heatmap was generated by the “complex heatmap“ R package with row-wise zScore transformation.

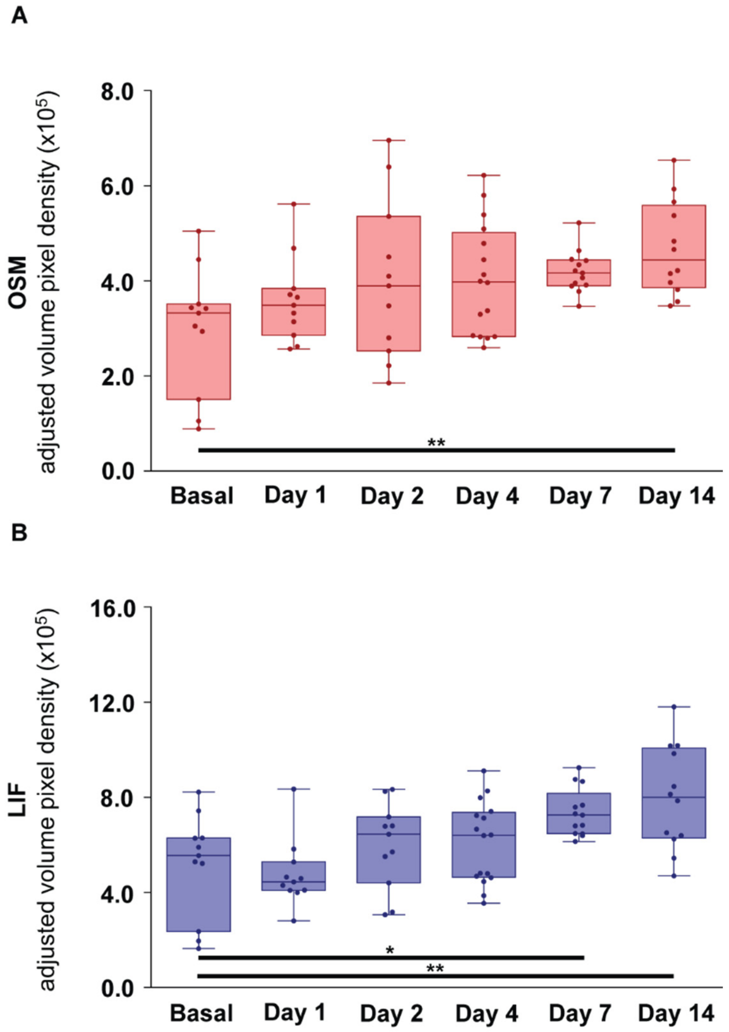

Supplementary Figure S2: Kinetic expression profile of OSM and LIF in serum of mice following the onset of myocardial infarction. Quantification of Immunoblot analysis of (**A**) OSM and (**B**) LIF in serum of mice under basal conditions and after myocardial infarction (MI) at indicated time points (n=11 at basal, n=11 at day–1, n=11 at day–2, n=16 at day–4, n=13 at day–7 and n=12 at day 14 post–MI). Semi–quantitative analysis of immunoblots is based on the adjusted mean volume pixel density of bands. Statistical analysis was performed by one–way ANOVA. Asterisks indicate Bonferroni post–test significances between distinct time points with * P<0.05 and ** P<0.01. Data are presented as mean ± sem.

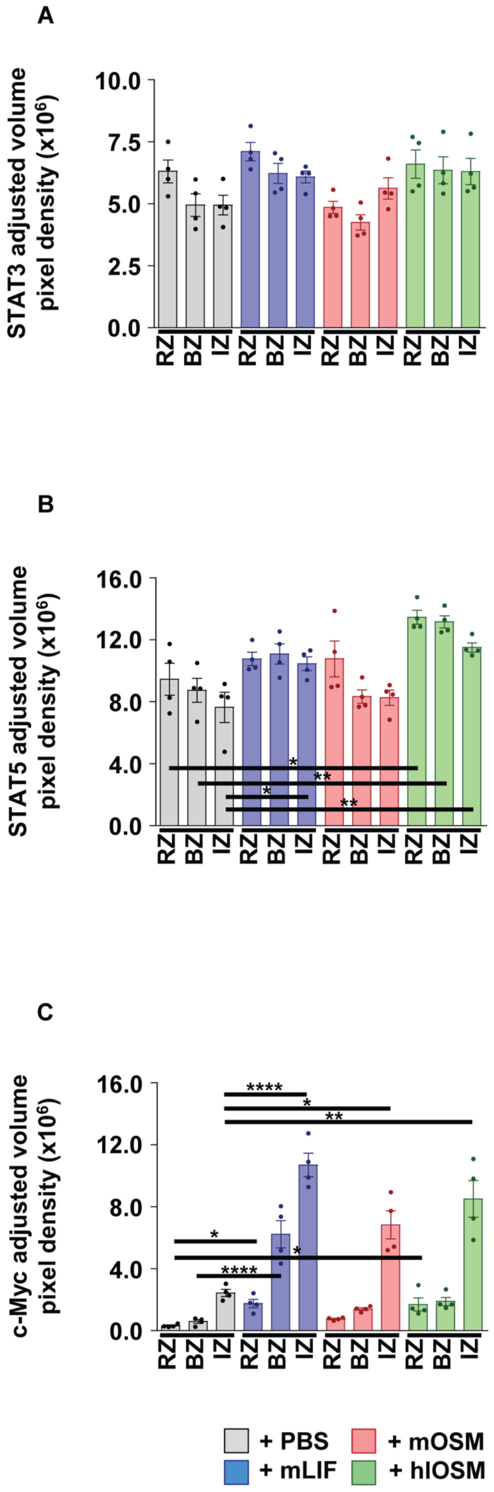

Supplementary Figure S3: Expression of STAT3, STAT5 and c–Myc at distinct sites of the myocardium after post–infarction administration of mLIF, mOSM and hlOSM. Semi–quantitative analysis of Immunoblot analysis of (A) STAT3 (B) STAT5 and (C) c–Myc in remote zone (RZ), border zone (BZ) and infarction zone (IZ) of mice (n=4 for all groups). Representative Immunoblots for STAT3, STAT5 and c–Myc are shown in Figure 5. Semi–quantitative analysis of immunoblots is based on the adjusted mean volume pixel density of bands. Statistical analysis was performed by one–way ANOVA. Asterisks indicate Bonferroni post–test significances between cytokine– and PBS–treated mice in RZ, BZ and IZ with * $P<0.05$, ** $P<0.01$ and **** $P<0.0001$. Data are presented as mean ± sem.

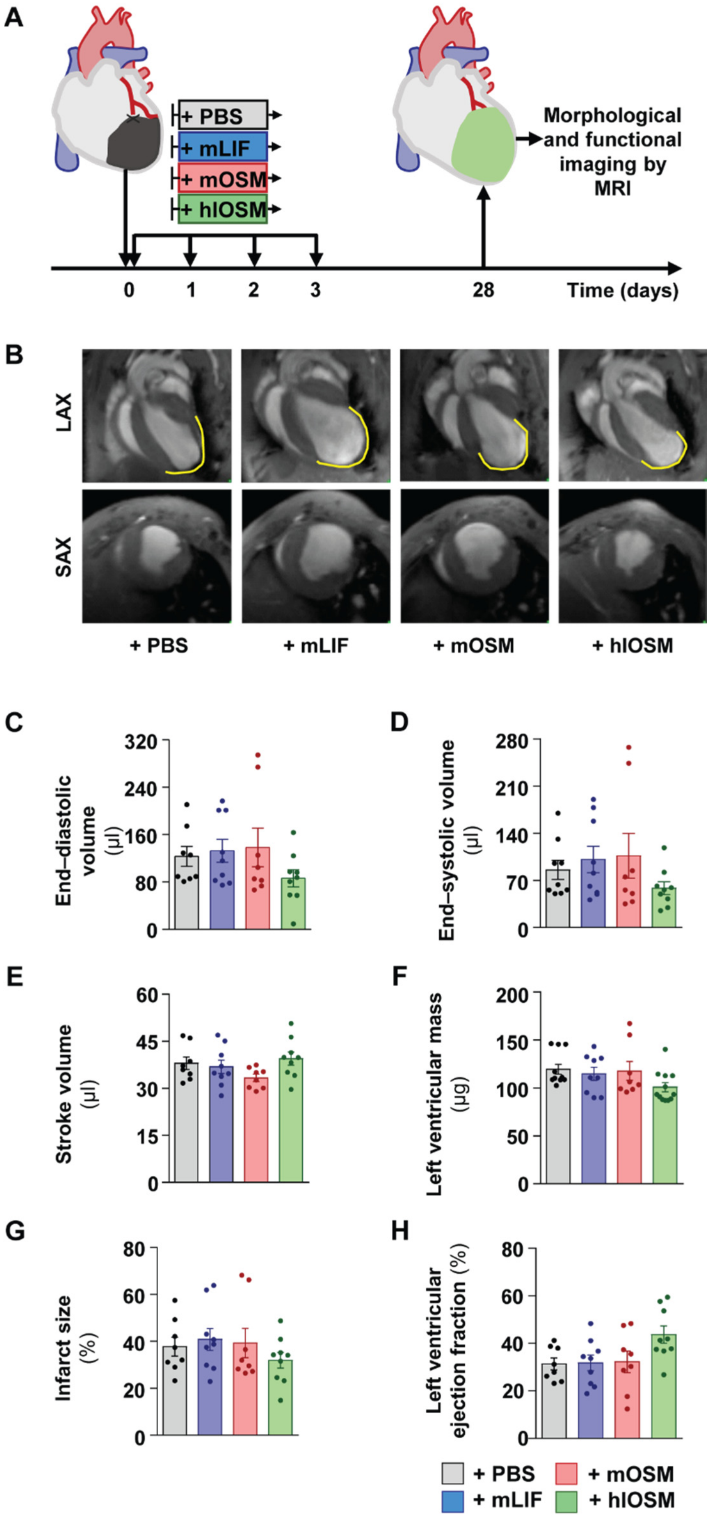

Supplementary Figure S4: Morphological and functional characterisation of hearts upon post–infarction administration of mLIF, mOSM and hIOSM by conventional magnetic resonance imaging. (A) Schematic illustration of morphological and functional characterization of hearts upon post–infarction administration of mLIF, mOSM and hIOSM by conventional magnetic resonance imaging at day 28 post–MI. (**B**) Representative long-axis (LAX) and short-axis (SAX) magnetic resonance frames in end-diastolic phase for each group. The semitransparent yellow line highlights infarcted areas. Column scatter plots of (**C**) end-diastole, (**D**) end-systolic and (**E**) stroke volumes, (**F**) lent ventriculas mass, (**G**) infarct size and (**H**) ejection fraction of the left ventricle for each group (n=4). Data are presented as mean ± sem.

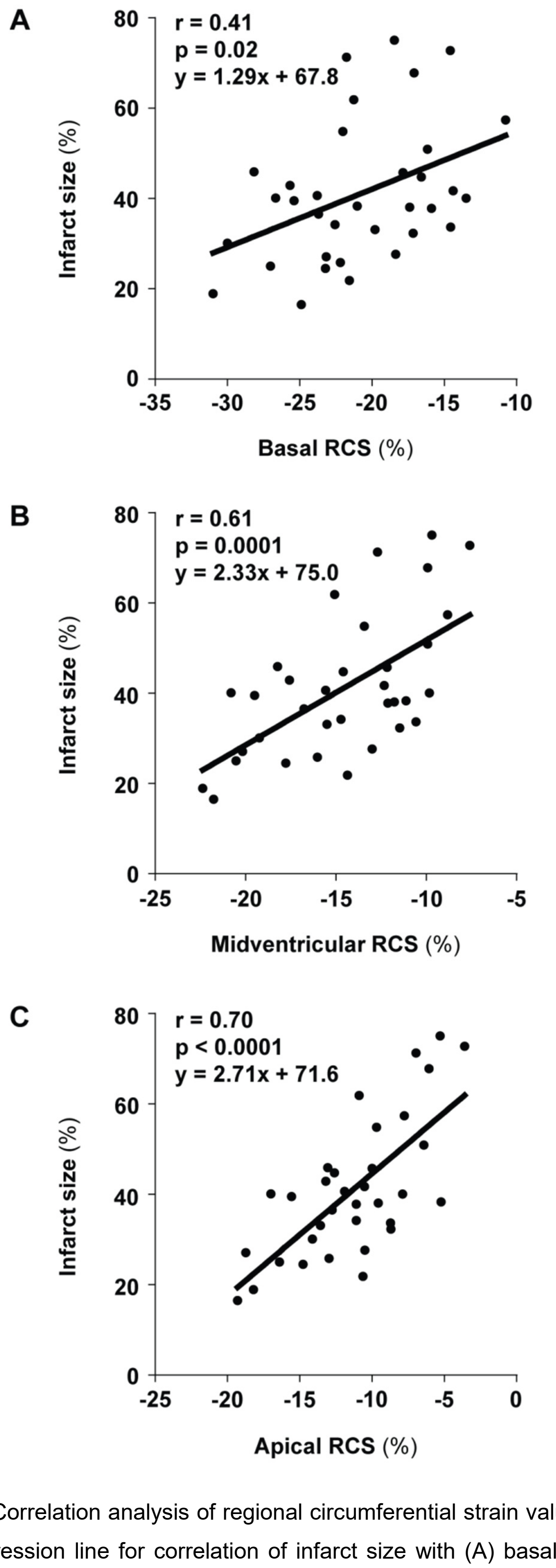

Supplementary Figure S5: Correlation analysis of regional circumferential strain values with infarct size. Scatter plots with corresponding regression line for correlation of infarct size with (A) basal, (B) midventricular and (C) apical circumferential strain.
